# Boosting Photovoltaic Performance of Dye-Sensitized Solar Cells Using Silver Nanoparticle-Decorated N,S-Co-Doped-TiO_2_ Photoanode

**DOI:** 10.1038/srep11922

**Published:** 2015-07-06

**Authors:** Su Pei Lim, Alagarsamy Pandikumar, Hong Ngee Lim, Ramasamy Ramaraj, Nay Ming Huang

**Affiliations:** 1 Low Dimensional Materials Research Centre, Department of Physics, Faculty of Science, University of Malaya, 50603 Kuala Lumpur, Malaysia; 2 Department of Chemistry, Faculty of Science, Universiti Putra Malaysia, 43400 UPM Serdang, Selangor, Malaysia; 3 Functional Device Laboratory, Institute of Advanced Technology, Universiti Putra Malaysia, 43400 UPM Serdang, Selangor, Malaysia; 4 Department of Physical Chemistry, School of Chemistry, Centre for Photoelectrochemistry Madurai Kamaraj University, Madurai-625 021, India

## Abstract

A silver nanoparticle-decorated N,S-co-doped TiO_2_ nanocomposite was successfully prepared and used as an efficient photoanode in high-performance dye-sensitized solar cells (DSSCs) with N719 dye. The DSSCs assembled with the N,S-TiO_2_@Ag-modified photoanode demonstrated an enhanced solar-to-electrical energy conversion efficiency of 8.22%, which was better than that of a DSSC photoanode composed of unmodified TiO_2_ (2.57%) under full sunlight illumination (100 mWcm^−2^, AM 1.5 G). This enhanced efficiency was mainly attributed to the reduced band gap energy, improved interfacial charge transfer, and retarded charge recombination process. The influence of the Ag content on the overall efficiency was also investigated, and the optimum Ag content with N,S-TiO_2_ was found to be 20 wt%. Because of the enhanced solar energy conversion efficiency of the N,S-TiO_2_@Ag nanocomposite, it should be considered as a potential photoanode for high-performance DSSCs.

Renewable energy sources have become important approaches for gaining independence from fossil fuels. Utilizing solar energy is certainly one of the most viable ways to solve the world’s energy crisis. Dye-sensitized solar cells (DSSCs) have emerged as promising candidates for harnessing solar power because of their low cost, flexibility, ease of production, relatively high energy conversion efficiency, and low toxicity to the environment^1^. Several efforts have been made to fabricate highly efficient DSSCs by the introduction of novel components such as electrolytes, dyes, counter electrodes, and semiconductor photoanodes. Among these DSSC components, the photoanode plays a vital role in determining the DSSC performance. So far, a titanium dioxide (TiO_2_) is one of the most commonly used photoanode materials, and it is promising material for a DSSC because of its low cost, ease of fabrication, relatively high energy conversion efficiency, high specific surface area, and non-toxicity. However, the major limitation associated with using TiO_2_ as a photoanode is its random electron transport, which will cause the electron–hole recombination process and hence hinder the cell performance^2^,^3^. In order to solve this serious issue, designing a photoanode with an efficient charge transport pathway from the photoinjected carriers to the current collector seems to be a possible alternative to enhance the performance of DSSCs. With this aim, surface modifications of TiO_2_ with metal doping, semiconductor coupling, and hybridization with a carbon material has have been attempted and shown better results^4^,^5^,^6^,^7^.

The modification of TiO_2_ with a noble metal such as gold (Au)^8^, silver (Ag)^9^, or platinum (Pt)^10^ has been actively reported for the photoanodes in DSSC applications to prevent the recombination of the photogenerated electron–hole pairs and improve the charge transfer efficiency. In the present decade, Ag nanoparticle-modified TiO_2_ has been applied as a photoanode with the aim of improving the efficiency of a DSSC. The Ag nanoparticles play dual roles in the DSSC performance, including the enhancement of the absorption coefficient of the dye and optical absorption due to surface plasmonic resonance^11^,^12^,^13^. Moreover, they act as an electron sink for photoinduced charge carriers, improve the interfacial charge transfer process, and minimize the charge recombination, thereby enhancing the electron transfer process in a DSSC^14^,^15^,^16^. Hence, the performance of a DSSC with Ag@TiO_2_ plasmonic nanocomposite material-modified photoanodes has been actively investigated^14^,^15^,^16^. On the other hand, to improve the light absorption of a TiO_2_ material in the visible region, non-metals such as nitrogen (N), carbon (C), sulfur (S), and phosphorus (P) have been widely studied as dopants for TiO_2_^17^. Among these, nitrogen doped TiO_2_ possesses good photovoltaic properties and hence has received much attention due to the narrowing of the band gap and shift to the visible region^18^. Hence, the co-deposition of Ag on doped TiO_2_ showed enhanced photovoltaic properties due to the synergistic effect between the Ag and doped TiO_2_^19^,^20^.

In the present investigation, we successfully developed a facile route to synthesize N,S-TiO_2_@Ag nanocomposite materials as a photoanode for high-performance dye-sensitized solar cells. The influence of the Ag content in the N,S-TiO_2_@Ag on the DSSC performance was also investigated. The introduction of plasmonic Ag nanoparticles on the N,S-TiO_2_ surface showed multifunctional properties, including a surface plasmon resonance effect, a reduction in the band-gap, an improved interfacial charge transfer, and the minimization of the charge recombination process as a result of the synergistic photovoltaic performance.

## Results and Discussion

### Morphological studies of N,S-TiO_2_@Ag nanocomposite

**Figure 1:**
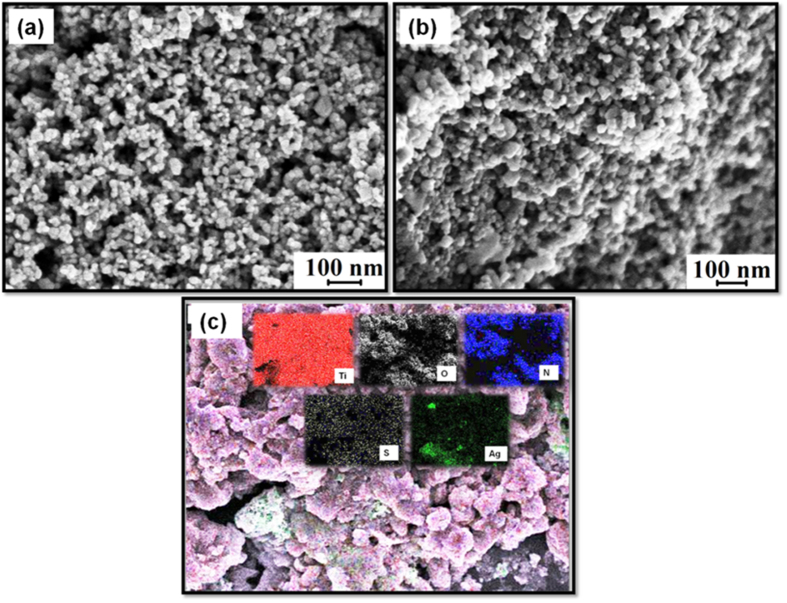
FESEM images of (**a**) N,S-TiO_2_, (**b**) N,S-TiO_2_@Ag and (**c**) element mapping of N,S-TiO_2_@Ag nanocomposite.

**Figure 2:**
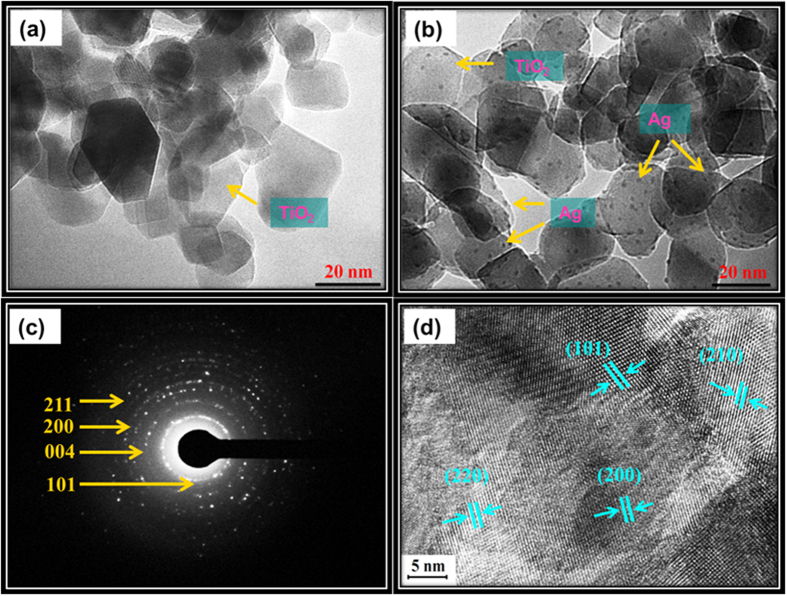
TEM images of (**a**) N,S-TiO_2_, (**b**) N,S-TiO_2_@Ag, (**c**) SAED pattern and (**d**) HRTEM image of N,S-TiO_2_@Ag.

Figure 1 shows the FESEM microscopic morphology of the as-prepared samples, and Fig. 1a shows an FESEM image of N,S-TiO_2_, which appeared to be spherical, with a uniform size. Upon the addition of Ag nanoparticles, no significant change in the morphology was observed for the N,S-TiO_2_@Ag film (Fig. 1b). An energy dispersive X-ray spectrometry (EDS) mapping analysis of the N,S-TiO_2_@Ag thin film also confirmed that the N,S-TiO_2_@Ag nanocomposite, composed of Ag, N, and S elements, was dispersed on the surface of the TiO_2_ nanoparticles with Ti and O signals (Fig. 1c). Further, TEM images of the N,S-TiO_2_ were also recorded and are shown in Fig. 2a. The TEM images show that the N,S-TiO_2_ nanoparticles are spherical in shape, with a TiO_2_ particle size range of 20–25 nm. Figure 2b clearly shows that small spherical Ag nanoparticles with a particle size range of 2–5 nm were well distributed and decorated on the surface of the N,S-TiO_2_ nanoparticles. Figure 2c depicts the selected area electron diffraction (SAED) pattern of the N,S-TiO_2_@Ag nanocomposite. The pattern clearly reveals bright concentric rings, which were due to the diffraction from the (211), (200), (004), and (101) planes of the anatase TiO_2_. In the HRTEM image of the N,S-TiO_2_@Ag (Fig. 2d), lattice fringes with d-spacing values of 3.51 Ǻ, 1.34 Ǻ, and 1.89 Ǻ were observed, which corresponds to the (101), (220), and (200) planes of the anatase TiO_2_, respectively; while the inter-planar d-spacing value of 2.05 Ǻ was assigned to the (210) plane of rutile TiO_2_.

### Crystalline studies of N,S-TiO_2_@Ag nanocomposite

The XRD pattern obtained for the N,S-TiO_2_@g is shown in Fig. S1, along with those of TiO_2_ and N,S-TiO_2_ for comparison. The TiO_2_, N,S-TiO_2_, and N,S-TiO_2_@Ag films were composed of mixed anatase and rutile phases, which agreed with reference patterns JCPDS 83-2243 and 21-1276, respectively. The diffraction peaks observed at 25.4°, 38.4°, 48.2°, and 74.5° corresponded to the anatase phase of TiO_2_ and were assigned to the (101), (004), (200), and (215) crystallographic planes. In contrast, the peaks at 54.09°, 63.2°, and 64.9° agreed well with the rutile phase of TiO_2_ and were assigned to the (220), (002), and (310) crystallographic planes. It was difficult to differentiate the Ag from the TiO_2_ signals because of the overlapping of the Ag signal with the rutile plane in the N,S-TiO_2_@Ag nanocomposite. The XRD patterns clearly confirmed the anatase and rutile phases were retained without a phase change after the nitrogen and sulfur doping and the deposition of Ag nanoparticles. The additional peaks were due to the ITO substrate.

**Figure 3:**
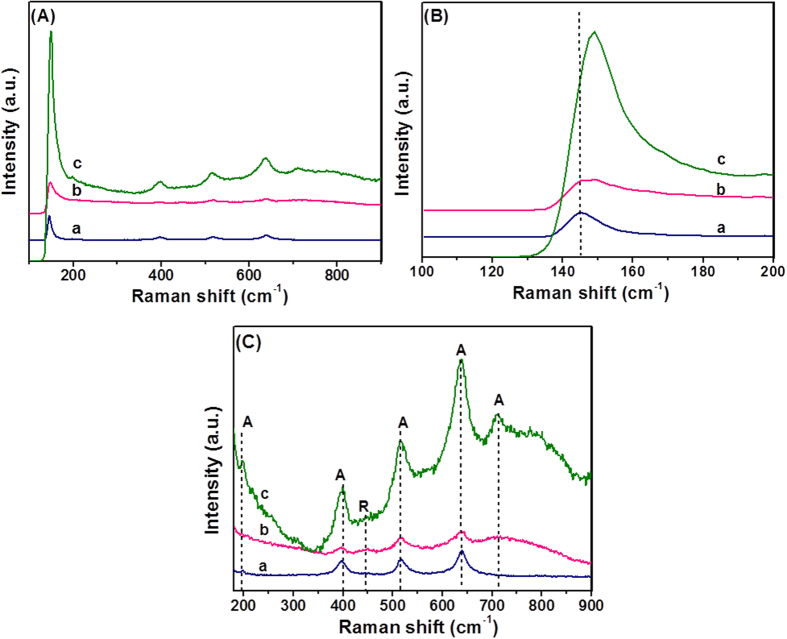
Raman spectra of (**a**) TiO_2_, (**b**) N,S-TiO_2_ and (**c**) N,S-TiO_2_@Ag in different frequency regions. A: 100–900 cm^−1^, B: 100–200 cm^−1^ and C: 180–900 cm^−1^ separately given for better clarity of the anatase and rutile phase TiO_2_.

To further evaluate the phases in the prepared films, Raman studies were performed in the range of 100–1000 cm^−1^, and the results are shown in Fig. 3a. The values of 147.48, 199, 395.28, 515.74, 639.25, and 710.18 cm^−1^ were due to the anatase phase TiO_2_^21^,^22^. The rutile TiO_2_ phase was observed at 444 cm^−1^ 21,22. This clearly indicated that the TiO_2_ and N,S-TiO_2_ nanoparticles contained a mixture of the anatase and rutile phases. The Raman signals of TiO_2_ did not change after adding nitrogen, which indicated that no phase transition occurred. However, no signals related to Ag particles were identified for the samples because of the relatively low concentration of Ag loaded onto the TiO_2_ and its weak Raman scattering power. An interesting observation was that the peak intensities increased with the deposition of Ag, while the position of the Raman signal remained the same. This indicates that there was an interaction between the Ag and TiO_2_ that affected the Raman resonance of TiO_2_^23^,^24^. This observation shows that the deposition of Ag on TiO_2_ and N,S-TiO_2_ does not cause any phase transition, but may bring about an electronic environment change in the surroundings^21^,^22^.

### XPS studies of N,S-TiO_2_@Ag nanocomposite

**Figure 4:**
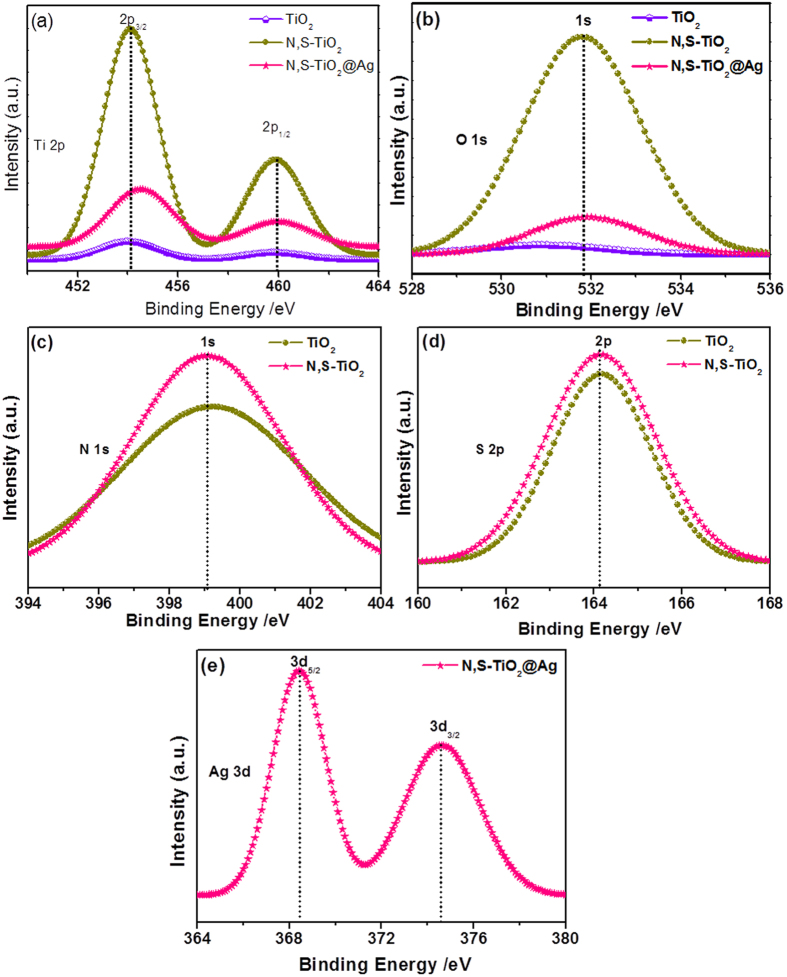
XPS spectra of TiO_2_, N,S-TiO_2_ and N,S-TiO_2_@Ag and their corresponding (**a**) Ti 2p (**b**) O 1s (**c**) N 1s (d) S 2p and (**e**) Ag 3d core-level spectra.

The XPS spectra of the TiO_2,_ N,S-TiO_2_, and N,S-TiO_2_@Ag were recorded in order to analyze and determine the chemical composition and identify the chemical states of the N, S, Ti, O, and Ag elements, and are shown in Fig. 4. Figure 4a shows the Ti 2p spectra for the samples, in which two peaks are observed at 454.1 and 460 eV, corresponding to the binding energies of Ti 2p_3/2_ and Ti2 p_1/2_ core levels due to the presence of the Ti(IV) state. Figure 4b shows the O1s spectra of TiO_2_ and Ag@TiO_2_, and the binding energy of the O 1s state of the samples was located at 531.1 eV, which was assigned to the bulk oxides (O^2−^) in the P25 lattice. The N 1s peak at 399.2 eV was assigned to the anionic N-doping, where O was replaced by N atoms involving substitutional N-doping, and the peak at 398.4 eV was also due to anionic N-doping but incorporated in the TiO_2_ as an N–Ti–O structural feature^25^−^27^ (Fig. 4c). Figure 4d shows the corresponding high-resolution XPS spectrum of the S 2p region, and the binding energy peak that appeared at around 164.1 eV corresponds to the Ti–S bond due to the fact that sulfur atoms replaced some of the oxygen atoms on the TiO_2_ surface^28^,^29^. The binding energies found for the Ag 3d_5/2_ and Ag 3d_3/2_ levels were 368.4 and 374.5 eV, respectively (Fig. 4e), with a peak separation of 6 eV due to the metallic silver. The XPS analysis provided support for the existence of elements such as Ti, O, N, S, and Ag in the nanocomposite materials.

### Optical properties of N,S-TiO_2_@Ag nanocomposite

The optical absorption properties of the TiO_2_, N,S-TiO_2_, and N,S-TiO_2_@Ag were studied by recording the UV–visible absorption spectra, which are shown in Fig. S2(A). It can be seen that the absorption edge of TiO_2_ was red shifted after the doping with N and S. The further deposition of Ag nanoparticles on the surface of the N,S-TiO_2_ significantly influenced the visible light absorption, and an additional peak was also observed at 517 nm. The enhanced absorption in the visible region was due to the surface plasmon resonance (SPR), which was aroused by the collective oscillation of electrons in the Ag nanoparticles stimulated by optical excitation^30^. The band-gap energy (E_bg_) values of the TiO_2_, N,S-TiO_2_, and N,S-TiO_2_-Ag were calculated using the well-known Tauc’s plot method^31^. The calculated E_bg_ values of TiO_2_, N,S-TiO_2_, and N,S-TiO_2_@Ag were 3.36, 3.20, and 2.90 eV, respectively (Fig. S2(B–D)). A remarkable reduction in the E_bg_ value of N,S-TiO_2_ was observed due to the substitution of oxygen by nitrogen and sulfur in the TiO_2_ lattice. The mixing of the N 2p and S 2p states with O 2p states resulted in a band gap narrowing of TiO_2_^17^. When Ag nanoparticles were added to the N,S-TiO_2_, the E_bg_ value was further decreased. This was due to the free electron properties being exhibited with a downward shift in the conduction band and upward shift in the valence band, which then led to a decrease in the E_bg_ value^31^,^32^.

**Figure 5:**
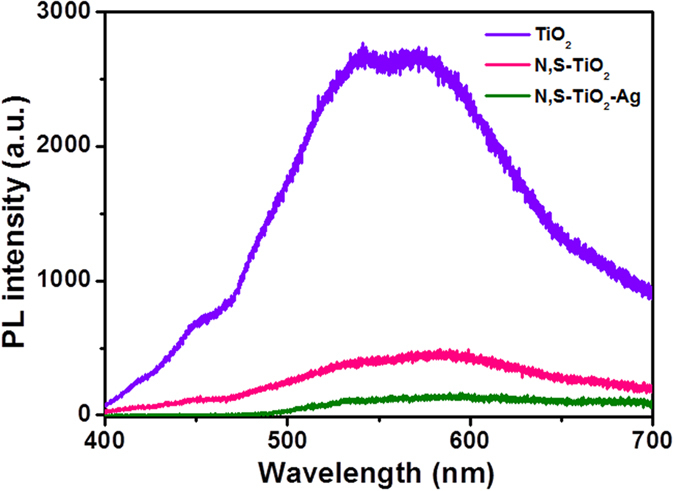
Photoluminescence spectra of (**a**) TiO_2_, (**b**) N,S-TiO_2_ and (**c**) N,S-TiO_2_@Ag.

Understanding the charge recombination process of a semiconductor is crucial because it significantly influences the photoelectrochemical properties and DSSC performance. Photoluminescence (PL) is a suitable tool to study the efficiency of charge carrier trapping, migration, and transfer, and to understand the fate of electron–hole pairs in semiconductor particles because PL emissions result from the recombination of free carriers. The TiO_2_ will absorb the incident photons with sufficient energy equal to or higher than the band-gap energy, which will produce photoinduced charge carriers (h^+^…e^−^). In addition, the recombination of photoinduced electrons and holes releases energy in the form of PL emission spectra. Hence, a lower PL intensity indicates less charge recombination. The observed PL spectra of TiO_2_, N,S-TiO_2_, and N,S-TiO_2_-Ag are shown in Fig. 5. A broad peak with a maximum emission at around 580 nm can be observed for TiO_2_, N,S-TiO_2_, and N,S-TiO_2_-Ag. The TiO_2_ showed a higher PL intensity due to the rapid recombination of photoinduced charge carriers. The PL intensity decreased when N, S, and Ag were introduced to TiO_2_. This can mainly be attributed to the formation of a Schottky barrier at the Ag and TiO_2_ interface, which could act as an electron sink to efficiently prevent the electronhole recombination process^33^.

### Photovoltaic performance of N,S-TiO_2_@Ag photoanode-modified DSSCs

**Figure 6:**
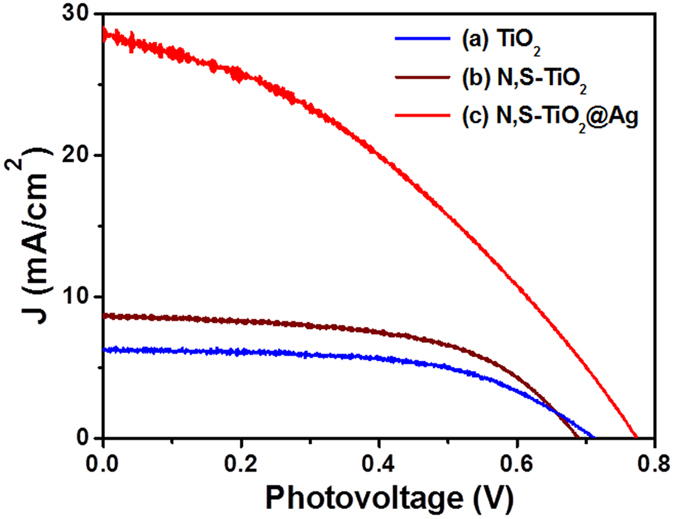
Photocurrent density-photovoltage (J-V) curves obtained for the (**a**) TiO_2_, (**b**) N,S-TiO_2_ and (**c**) N,S-TiO_2_@Ag photoanode modified DSSCs.

The photovoltaic performances of TiO_2_, N,S-TiO_2_, and N,S-TiO_2_@Ag photoanode-modified DSSCs under simulated solar irradiation of AM 1.5 G were studied by recording the J-V profiles, which are shown in Fig. 6. The corresponding photovoltaic parameters are summarized in Table 1. A significant boost in the short-circuit current up to 29.05 mA/cm^2^ can be observed from the J-V curve of N,S-TiO_2_@Ag compared to those of the DSSCs based on TiO_2_ (6.27 mA/cm^2^) and N,S-TiO_2_ (9.78 mA/cm^2^). The N,S-TiO_2_@Ag-based DSSC showed a conversion efficiency (*η* = 8.22%) that was 145% and 231% higher than those of the DSSCs based on N,S-TiO_2_ (*η* = 3.35%) and pure TiO_2_ (*η* *=* 2.57%). This significant improvement in the photovoltaic performance was due to the synergetic effect of the N, S, and Ag. The presence of N in the TiO_2_ successfully reduced the band gap and extended the absorption of TiO_2_ into the visible region. The existence of S facilitated the electron transfer process and consequently increased the forward reduction reaction. The electrons from the conduction band could transfer to the more electropositive S atoms, which were reduced from S^6+^ to S^4+^. The electrons were then transferred to the Ag to retain the system at S^6+^ 19,20. Moreover, the deposition of Ag onto the N,S-TiO_2_ resulted in a change in the Fermi energy level. A large number of the electrons accumulated on the surfaces of the Ag nanoparticles due to the surface plasmon resonance effect. The accumulation of these electrons on the Ag nanoparticles shifted the position of the Fermi level closer to the conduction band of TiO_2_^34^. The electrons in the Ag nanoparticles that were excited due to the SPR effect were transferred to the conduction band of the TiO_2_ and collected by the current collector (ITO), which improved the photocurrent under irradiation in the visible region^35^. In this way, the photovoltaic performance significantly improved with the enhancement of absorption in the visible region. The synergetic effect of Ag and S as a redox couple played a potential role in the enhanced DSSC performance. The controlled experiments also carried out for N,S-TiO_2_@Ag sample without dye loading and the result is shown (Fig. S3). The photocurrent response of ~0.15 mA/cm^2^ was observed due to the plasmonic excitation of Ag nanoparticles and it suggests that coupling of Ag nanoparticles with N,S-TiO_2_ in the presence of N719 sensitizer can produce synergistic effect in DSSC.

### Influence of Ag content on photovoltaic performance of N,S-TiO_2_@Ag photoanode-modified DSSCs

**Figure 7:**
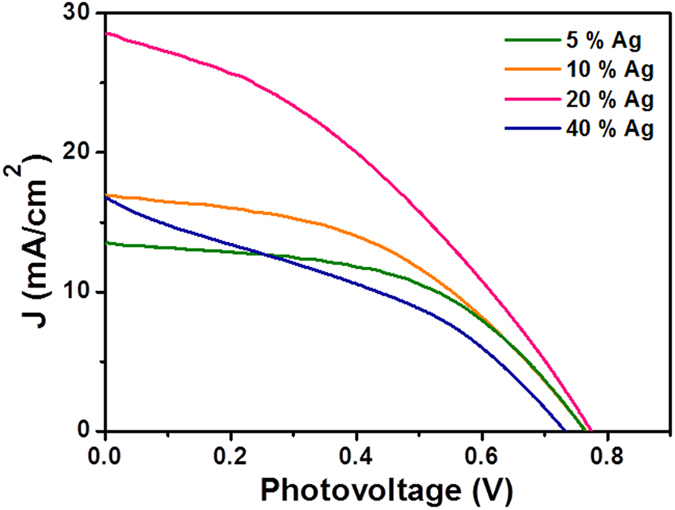
Photocurrent density-photovoltage (J-V) curves obtained for the N,S-TiO_2_@Ag nanocomposite modified photoanodes with different Ag content.

**Figure 8:**
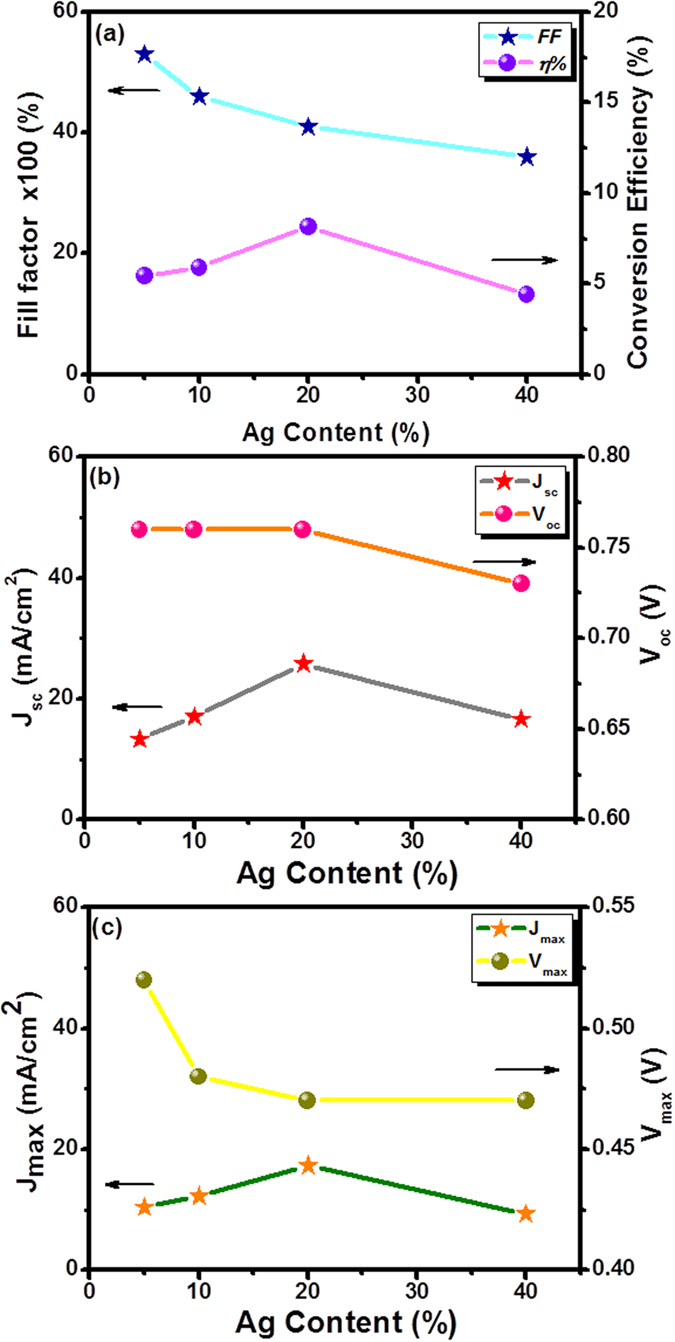
Plots of (**a**) Fill factor (FF) and power conversion efficiency (*η*), (**b**) short-circuit current density (J_sc_) and open-circuit voltage (V_oc_) and (**c**) maximum photocurrent density (J_max_) and maximum photovoltage (V_max_) with different Ag content.

The loading of the Ag content in the N,S-TiO_2_@Ag photoanode was varied to obtain a high-performance DSSC. The photocurrent density–photovoltage (J-V) curves were recorded for the N,S-TiO_2_@Ag nanocomposite-modified photoanodes with different Ag contents and are shown in Fig. 7. Their corresponding photovoltaic parameters were also evaluated and are listed in Table 2. The obtained conversion efficiency for the N,S-TiO_2_ photoanode without Ag showed a value of 3.39%, whereas N,S-TiO_2_ with 20% Ag showed an efficiency of 8.22%. Those with 5%, 10%, and 40% Ag showed conversion efficiencies of 5.42%, 5.90%, and 4.40%, respectively. The observed results clearly revealed that the conversion efficiency of the DSSC was increased with an increase in the Ag content in the photoanode until it reached a maximum of 20%, and a further increase in the Ag content eventually led to a decrease in the conversion efficiency (Fig. 8a and Table 2). The decrease in efficiency at a high Ag loading was due to the free standing/excess Ag in the composite, which could oxidize to Ag(I)^16^,^36^ and erode to the redox electrolyte^16^. The oxidation of the Ag would have acted as a new recombination center, thus reducing the number of charge carriers, which led to a decrease in the J_sc_ and V_oc_. Consequently, the overall conversion efficiency of the DSSC deteriorated. The J_sc_ was increased by an increase in the Ag content and attained a maximum of 29.05 mA/cm^2^ with 20% (Fig. 8b). Then, J_sc_ decreased to 16.65 mA/cm^2^ with 40% Ag loading in the photoanode of the DSSC. It could also be seen that the V_oc_ trend was almost the same, within the range of ~0.69–0.75 V. The J_max_ and V_max_ values of the device also followed trends similar to those of J_sc_ and V_oc_ (Fig. 8c).

### Electrochemical behavior of N,S-TiO_2_@Ag photoanode-modified DSSCs

**Figure 9:**
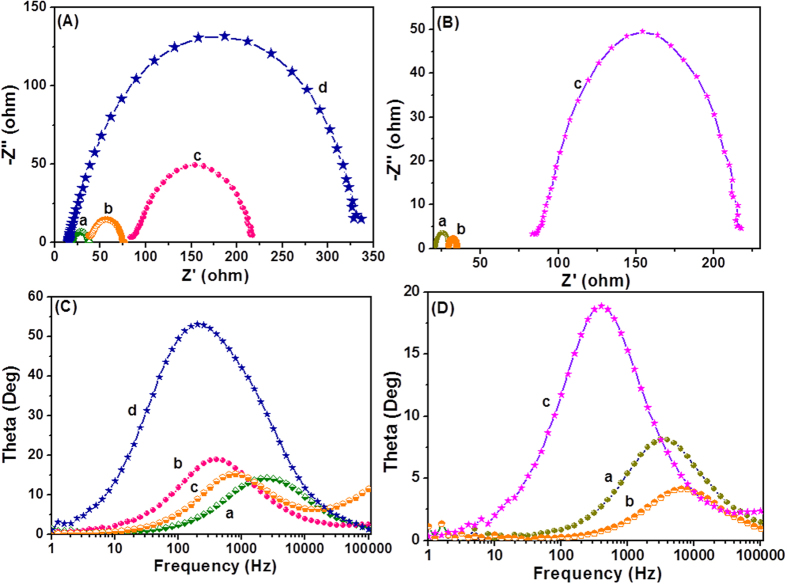
(**A**) Nyquist plot and (**C**) Bode phase plots obtained for N,S-TiO_2_@Ag nanocomposite modified photoanodes with (a) 5, (b) 10, (c) 20, and (d) 40% of Ag content. (**B**) Nyquist plot and (**D**) Bode phase plots of (a) TiO_2_, (b) N/S-TiO_2_ and (c) N,S-TiO_2_@Ag.

For the interfacial charge transfer process within the fabricated DSSC with the N,S-TiO_2_@Ag photoanode, the electrochemical impedance spectra (EIS) were recorded in the frequency range between 0.01 Hz and 100 KHz, and are shown in Fig. 9. Well-defined semicircles in the middle frequency region for the DSSCs with N,S-TiO_2_@Ag nanocomposite-modified photoanodes with different Ag contents were observed in the Nyquist plots (Fig. 9(A)). The intersection of the high-frequency semicircle at the real axis represents the equivalent series resistance of the device (R_s_); the arc in the middle frequency range between 1 and 1000 Hz represents the charge transfer resistance (R_ct_) between the dye-adsorbed photoanode and electrolyte interface^2^,^37^. The R_s_ values varied because different concentrations of Ag used in the photoanode. An increase in the R_ct_ value of the N,S-TiO_2_@Ag-based photoanode was observed with the increasing Ag content. Thus, a photoanode that contained a high amount of Ag with a higher R_ct_ value corresponded to an ineffective electron transfer process between the photoanode and electrolyte interface. The changes in R_s_ and R_ct_ could mainly be attributed to the changes in the Ag content of the photoanode, which contributed the most to the internal impedance. The Nyquist plot for different types of photoanodes is shown in Fig. 9(B). As demonstrated in the Nyquist plot, both the real (*Z*’) and imaginary (*Z*”) parts of the total impedance, as well as the R_ct_ value, increased in the order N,S-TiO_2_ < TiO_2_ < N,S-TiO_2_@Ag, which showed the crucial dependence of the charge transport on the dopants (N and S) and Ag incorporation .The R_s_ value also increased with the addition of N, S, and Ag. Therefore, the origin of the higher J_sc_ in N,S-TiO_2_@Ag is expected to have arisen from the device resistance (R_s_), R_ct_, and charge transport dynamics determined by the electron lifetime (*τ*_n_). Based on the Bode phase plots (Fig. 9(C,D)), the frequency was apparently shifted to the lower frequency region with the addition of N, S, and Ag. The maximum frequencies (*ω*_max_) in the middle frequency region of the Bode plots for TiO_2_ and N,S-TiO_2_@Ag were 792.44 Hz and 316.23 Hz, respectively. Since *ω*_max_ is inversely associated with the electron lifetime *τ*_n_ = 1/(2*πf*)^38^,^39^, the decrease in *ω*_max_ indicated a reduced rate for the charge-recombination process in the DSSC. Hence, electrons with longer *τ*_n_ values were prevented from recombining, characterized by a larger R_ct_.

**Figure 10:**
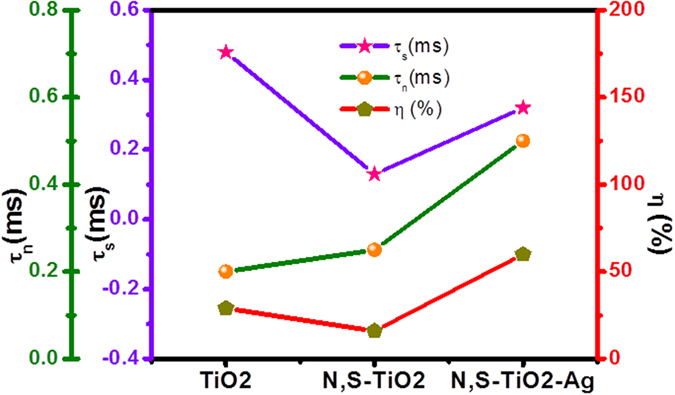
Electron lifetime, electron transport time and charge collection efficiency of TiO_2_, N,S-TiO_2_ and N,S-TiO_2_@Ag photoanode based DSSCs.

Furthermore, Table 3 and Fig. 10 summarize the results obtained from the Nyquist plot. The N,S-TiO_2_@Ag photoanode exhibited a faster electron transport time (*τ*_s_ = R_s_*C_μ_)^38^,^40^,^41^ than TiO_2_. Hence, its electron lifetime (*τ*_n_ = R_ct_*C_μ_))^38^,^40^,^41^ was significantly increased, and they were prevented from recombining. The photovoltaic performance of the DSSC was clearly reflected by the charge collection efficiency (η_c_) derived from η_c_ = (1+R_s_/R_ct_)^−1^ 38,40,41. Eventually, the charge collection efficiency was significantly increased upon the addition of N, S, and Ag. Therefore, we can conclude that as a result of the longer *τ*_n_ and larger R_ct_, the devices fabricated using N,S-TiO_2_@Ag showed improved J_sc_ values compared to the TiO_2_ and N,S-TiO_2_ photoanode-based DSSCs.

The electrocatalytic activity of the Pt counter electrode was investigated by cyclic voltammetry (CV) technique in 10 mM LiI, 1 mM I_2_ and 0.1 M LiClO_4_ acetonitrile solution. The observed more negative cathodic peak due to the reduction of tri-iodide and the anodic peak corresponds to the reverse reaction^42^. Further, the role of Pt counter electrode on the photovoltaic performance of DSSC was studied by electrochemical impedance spectroscopy (EIS) analysis (Fig. S4(b and c)). The charge transfer resistance (R_CT_) of the electrode was calculated as 103.41 Ω from the half the real impedance of the high-frequency side semicircle and thus indicate the higher electrocatalytic activity of Pt counter electrode.

### Operation principle of N,S-TiO_2_@Ag photoanode-modified DSSCs

**Figure 11:**
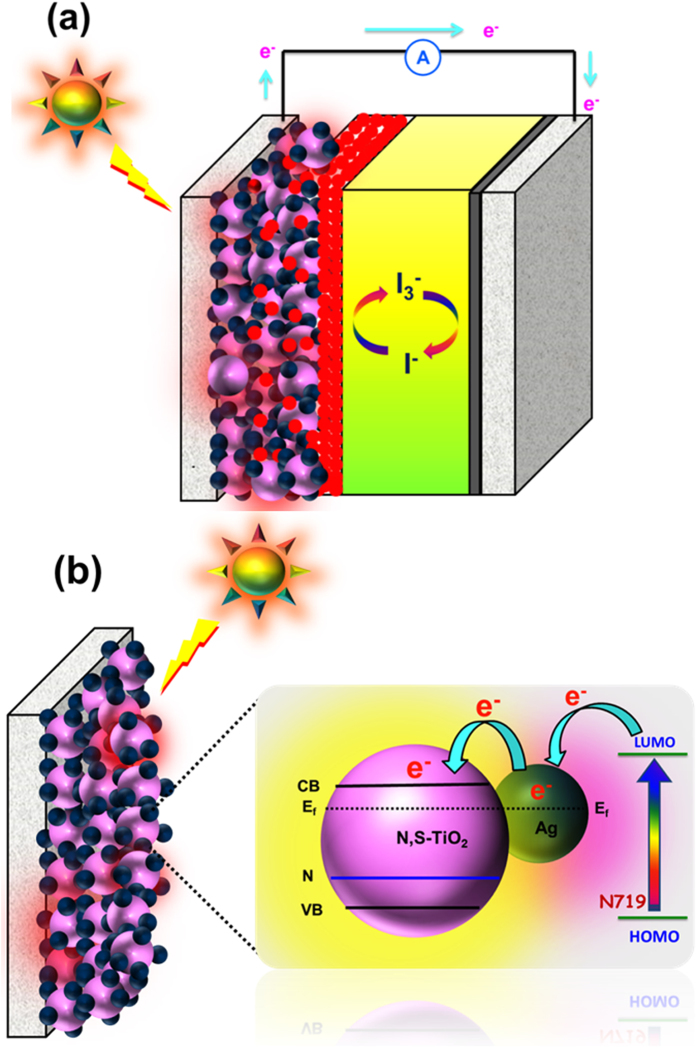
Schematic on (**a**) operation principles of DSSC containing N,S-TiO_2_@Ag photoanode and (**b**) photoinduced charge transfer process at the N,S-TiO_2_@Ag photoanode.

The operation principle of the N,S-TiO_2_@Ag nanocomposite photoanode-modified DSSC under illumination is shown in Fig. 11a. Upon illumination, the adsorbed dye molecules (N719) underwent photo-excitation. The excited electrons were injected into the conduction band of the N,S-TiO_2_ nanocomposite. The dye^+^ became oxidized by receiving electrons from the electrolyte through the redox system, and was regenerated. The electrolyte itself was regenerated *via* the platinum counter electrode by electrons passing through the external circuit. In our study, the deposition of Ag onto the surface of N,S-TiO_2_ not only acted as an electron sink for efficient charge transfer, but could also be used as a scattering element for plasmonic scattering to trap the light in the near field coupled with the dye molecules^43^. This will eventually improve the optical absorption of the dye, resulting in a significant photocurrent enhancement (Fig. 11b). In addition, the inclusion of Ag nanoparticles resulted in a change in the Fermi energy level. The electrons in the conduction band of the TiO_2_ could be effectively captured by the Ag until a Fermi level equilibrium was obtained and the charge recombination process was minimized, which improved the DSSC performance. The ideal property of Ag nanoparticles and the presence of N and S also successfully reduced the band gap and helped to shift the optical absorbance toward the visible region. The incorporation of N in the TiO_2_ lattice in the form of N-Ti-O partially converted the system from Ti^4+^ to Ti^3+^ and effectively contributed to the visible light absorption^44^,^45^. Furthermore, the existence of S also played a vital role in the photovoltaic performance, facilitated the electron transfer process, and consequently increased the forward reduction reaction. The Ag and S acted as co-catalysts and effectively minimized the electron recombination process. The attractiveness of the present photoanode is that it combines all of the photovoltaic fundamental criteria within its electronic environment.

## Conclusion

A simple approach to prepare Ag nanoparticle-deposited N,S-TiO_2_ *via* a chemical reduction method to fabricate a novel photoanode for a DSSC was demonstrated. The N,S-TiO_2_@Ag nanocomposite was characterized using UV–visible absorption, PL, Raman, XRD, XPS, SEM, EDAX, and TEM analyses. The DSSC fabricated with an N,S-TiO_2_@Ag-modified photoanode showed an enhanced solar-to-electrical energy conversion efficiency of 8.22% compared to the photoanode of a DSSC composed of unmodified TiO_2_ (2.57%) under simulated solar irradiation of 100 mWcm^−2^ with AM 1.5 G. The enhancement in the photovoltaic performance was mainly attributed to the plasmonic Ag nanoparticles, which enhanced the visible light adsorption as a result of their light harvesting property in the visible range due the surface plasmon effect. These Ag nanoparticles also significantly promoted interfacial charge transfer, which minimized the charge recombination process. The optimal Ag content in the N,S-TiO_2_ to obtain an efficient photoanode was found to be 20% Ag. In addition, the presence of the N and S dopants also helped to reduce the band gap, shift the optical absorbance toward the visible region, and also suppress the charge recombination. The synergetic effect of the Ag nanoparticles, surface plasmon effect, reduction of the band gap, and effective charge transfer ameliorated the photocurrent generation and conversion efficiency of the DSSC.

## Experimental Methods

### Materials

Titanium dioxide (P25) was purchased from Acros Organics. Silver nitrate (AgNO_3_) and Thiourea were purchased from Merck and Fluka, respectively. Sodium borohydride (NaBH_4_) was purchased from R&M chemicals. Indium tin oxide (ITO) conducting glass slides (7 Ω/sq) were purchased from Xin Yan Technology Limited, China. N719 (Ruthenizer 535-bisTBA) and iodolyte Z-100 were received from Solaronix.

### Materials characterization techniques

The crystalline phase of each sample was determined via X-ray diffraction (XRD; D5000, Siemens), using copper Kα radiation (λ = 1.5418 Å) at a scan rate of 0.02° s^−1^. The morphology of each film was examined using field emission scanning electron microscopy (Hitachi, SU 8000), transmission electron microscopy TEM (Hitachi, HT-7700), and high-resolution TEM (JEOL JEM-2100 F). The optical absorption properties in the spectral region of 190–900 nm were assessed using a Thermo Scientific Evolution 300 UV–vis spectrophotometer. The photoluminescence and Raman spectra were collected using a Renishaw inVia 2000 system with an argon ion laser emitting at 325 and 532 nm, respectively. X-ray photoelectron spectroscopy (XPS) measurements were performed using synchrotron radiation from beamline no. 3.2 at the Synchrotron Light Research Institute, Thailand.

### Synthesis of N,S-TiO_2_-Ag nanocomposite

The N,S-TiO_2_@Ag nanocomposite was prepared as follows. Initially, TiO_2_ and thiourea at a 1:1 weight ratio were mixed and ground for at least 30 min in a mortar. The mixture was then annealed in a furnace at 400 °C with a heating rate of 10 °C/min in air for 1 h. Subsequently, the pale yellow N,S-TiO_2_ was obtained. The N,S-TiO_2_@Ag nanocomposite was prepared using a chemical reduction method. A 500-mg quantity of N,S-TiO_2_ was added to an aqueous solution containing the AgNO_3_. The weight percentage of the AgNO_3_ to N,S-TiO_2_ varied from 0% to 5%, 10%, 20%, and 40%. Each mixture was vigorously stirred for 30 min at room temperature. The reduction of Ag^+^ was carried out by the drop-wise addition of a freshly prepared ice-cold NaBH_4_ solution until the color of the mixture changed. A yellowish green solution indicated the formation of the N,S-TiO_2_@Ag nanocomposite, after which and the solution continued to be stirred for another 30 min. The nanocomposite was collected and washed with distilled water and ethanol several times by centrifugation. Finally, the product was dried in an oven at 60 °C.

### Fabrication of N,S-TiO_2_@Ag-modified photoanode

The N,S-TiO_2_@Ag**-**modified photoanodes were fabricated using the following procedure. Initially, 300 mg of the N,S-TiO_2_@Ag nanocomposite was mixed in an ethanolic solution and stirred for 30 min. A 0.1 M quantity of TTIP was slowly introduced into the above reaction mixture and stirred until a homogenous solution was obtained. Finally, the N,S-TiO_2_@Ag nanocomposite was coated on a conducting side of the ITO using the doctor-blade technique. In order to obtain a stable photoanode, the film was dried at room temperature, sintered at 150 °C for 30 min in a muffle furnace, and then allowed to cool naturally to room temperature.

### Fabrication of DSSCs and evaluation of their performances

The prepared N,S-TiO_2_@Ag nanocomposite photoanodes were immersed into an ethanolic solution of 0.3 mM N719 (Ruthenizer 535-bisTBA) dye for 24 h at room temperature. The dye-adsorbed photoanode was withdrawn from the solution and immediately cleaned with ethanol. A platinum-sputtered ITO was placed on the dye-absorbed photoanode, and they were clamped firmly together. An electrolyte (Iodolyte Z-100, Solaronix) solution was introduced into the cell assembly by capillary action. A 150-W Xenon arc lamp (Newport, Model 69907) containing a simulated AM 1.5-G filter was used as a light source throughout the experiments. Prior to testing the photovoltaic parameter, an Avaspec-2048 fiber optic spectrophotometer was used to measure the light illumination intensity. The photocurrent signal measurements (J-V and J-T curves) were carried out with an active electrode area of 0.5 cm^2^ using a computer-controlled VersaSTAT 3 electrochemical workstation (Princeton Applied Research, USA).

## Additional Information

**How to cite this article**: Lim, S. P. *et al.* Boosting Photovoltaic Performance of Dye-Sensitized Solar Cells Using Silver Nanoparticle-Decorated N,S-co-doped-TiO_2_ Photoanode. *Sci. Rep.* **5**, 11922; doi: 10.1038/srep11922 (2015).

## Supplementary Material

Supplementary Information

## Figures and Tables

**Table 1:** Photovoltaic parameters of the fabricated DSSCs.

**Photoanode**	**J_sc_ (mA/cm^2^)**	**V_oc_ (V)**	**J_max_ (mA/cm^2^)**	**V_max_ (V)**	**FF**	**η (%)**
TiO_2_	6.27	0.70	5.25	0.49	0.59	2.57
N,S-TiO_2_	9.78	0.69	6.69	0.50	0.50	3.35
N,S-TiO_2_@Ag	29.05	0.77	17.88	0.46	0.37	8.22

The DSSCs performance was evaluated under 100 mW cm^−2^ simulated AM 1.5G solar light irradiation. J_sc_: Short-circuit current density; V_oc_: Open-circuit voltage; J_max_: Maximum photocurrent density; V_max_: Maximum photovoltage; FF: Fill factor; η: Power conversion efficiency. Area of the cell electrode was 0.5 cm^2^

**Table 2:** Photovoltaic parameters of the N,S-TiO_2_@Ag photoanode with various Ag content.

**Ag (%)**	**J_sc_(mA/cm^2^)**	**V_oc_ (V)**	**J_max_(mA/cm^2^)**	**V_max_(V)**	**FF**	**η (%)**
0	9.78	0.69	6.69	0.50	0.50	3.35
5	13.33	0.76	10.42	0.52	0.53	5.42
10	17.04	0.76	12.29	0.48	0.49	5.90
20	29.05	0.77	17.88	0.46	0.37	8.22
40	16.65	0.73	9.37	0.47	0.36	4.40

The DSSCs performance was evaluated under 100 mW cm^−2^ simulated AM 1.5G solar light irradiation. J_sc_: Short-circuit current density; V_oc_: Open-circuit voltage; J_max_: Maximum photocurrent density; V_max_: Maximum photovoltage; FF: Fill factor; η: Power conversion efficiency. Area of the cell electrode was 0.5 cm^2^.

**Table 3:** EIS analysis results of the fabricated DSSC.

**Photoanode**	**R_s_ (Ω)**	**R_ct_ (Ω)**	**C_μ_ (μF)**	**τ_s_ (ms)**	**τ_n_ (ms)**	**η_c_ (%)**
TiO_2_	21.01	8.91	7.10	0.48	0.20	29
N,S-TiO_2_	17.21	12.36	8.12	0.13	0.25	16
N,S-TiO_2_@Ag	15.34	20.28	6.23	0.32	0.50	60

The electrochemical impedance spectra (EIS) were recorded at an applied bias of −0.7 V in the frequency range between 0.01 Hz and 100 KHz. R_s_: Devise resistance; R_ct_: Charge transfer resistance; C_μ_: chemical capacitance; τ_s:_ Electron transport time; τ_n_: Electron lifetime; η_c_: charge collection efficiency.
