# Supplementary material for: Boosting Photovoltaic Performance of Dye-Sensitized Solar Cells Using Silver Nanoparticle-Decorated N,S-Co-Doped-TiO2 Photoanode
Source: Sci Rep. 2015 Jul 6;5:11922. doi: 10.1038/srep11922 (PMC4491728; doi:10.1038/srep11922)
Supplement: Supplementary Information [file srep11922-s1.pdf]

Boosting Photovoltaic Performance of Dye-Sensitized Solar Cells Using Silver Nanoparticle-Decorated N,S-co-doped-TiO_2 Photoanode

Su Pei Lim[1], Alagarsamy Pandikumar [1]*, Hong Ngee Lim [2,3*], Ramasamy Ramaraj[1,4], Nay Ming Huang [1]*

[1]Low Dimensional Materials Research Centre, Department of Physics, Faculty of Science, University of Malaya, 50603 Kuala Lumpur, Malaysia
[2]Department of Chemistry, Faculty of Science, Universiti Putra Malaysia, 43400 UPM Serdang, Selangor, Malaysia
[3]Functional Device Laboratory, Institute of Advanced Technology, Universiti Putra Malaysia, 43400 UPM Serdang, Selangor, Malaysia.
[4]Department of Physical Chemistry, School of Chemistry, Centre for Photoelectrochemistry Madurai Kamaraj University, Madurai-625 021, India.
*Corresponding author's: pandikumarinbox@gmail.com (Pandikumar A)
huangnayming@um.edu.my (Huang N.M.)
janet_limhn@science.upm.edu.my (Lim H.N.)

Supplementary Information

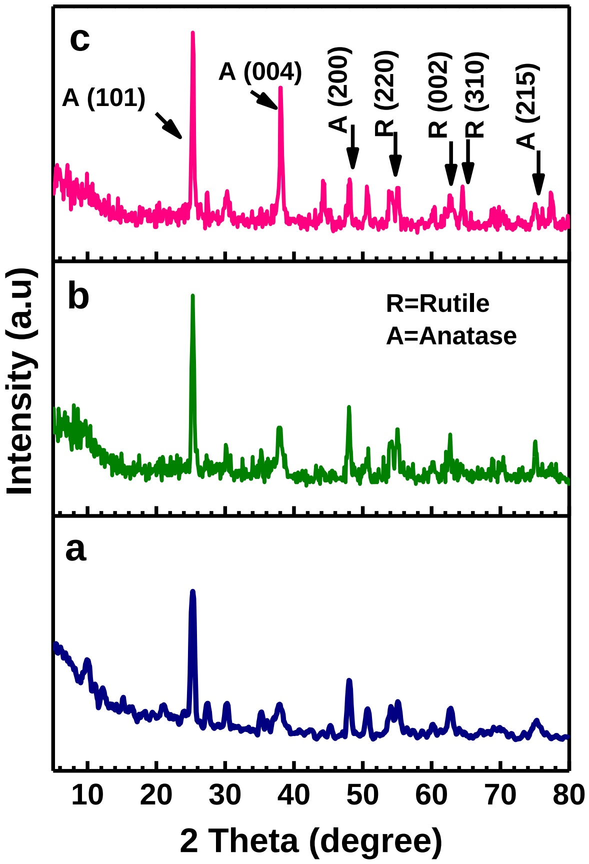

Fig. S1. XRD patterns of (a) TiO_2, (b) N,S-TiO_2 and (c) N,S-TiO_2@Ag.

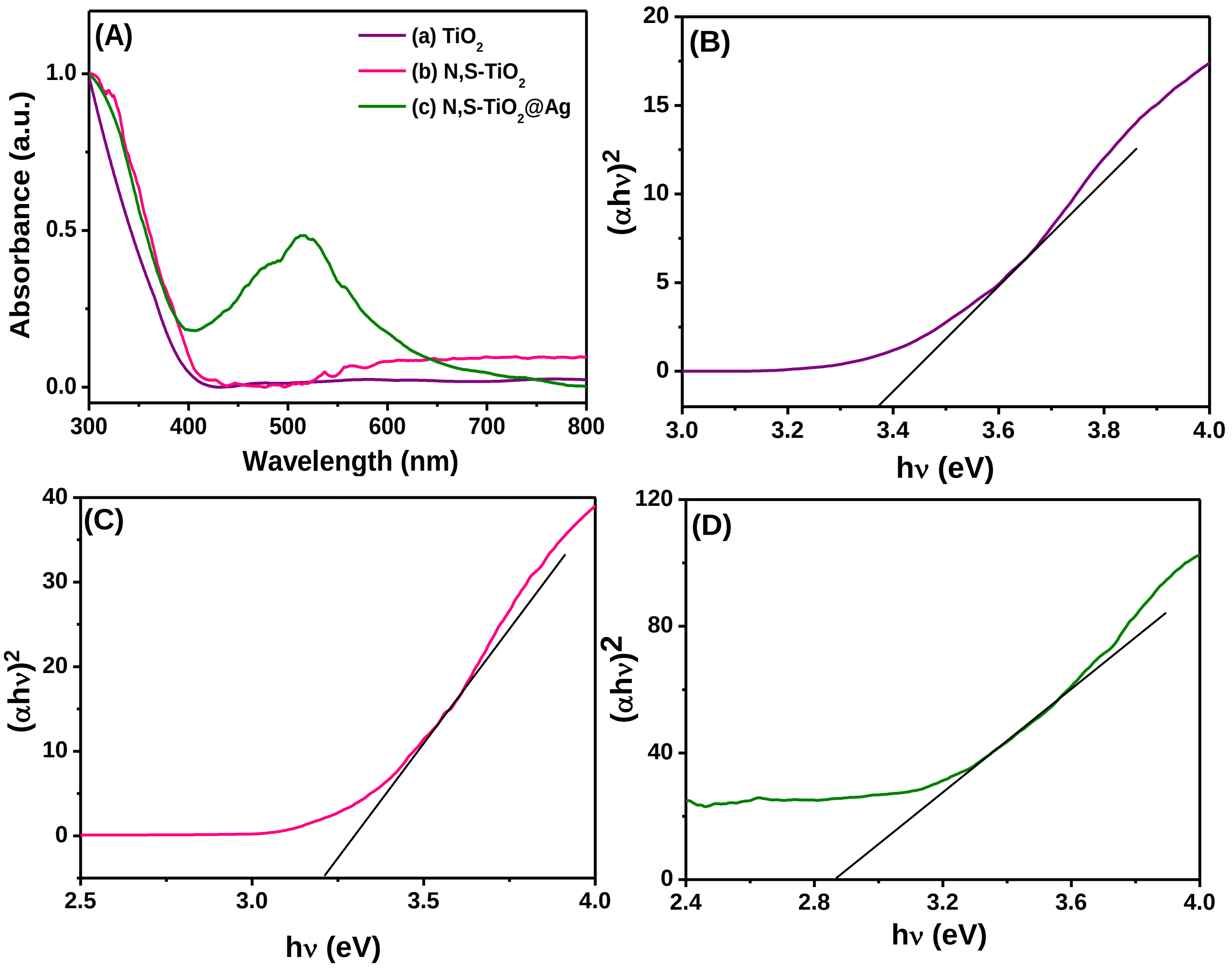

Fig. S2 (A) UV-Visible absorption spectra of the (a) TiO_2, (b) N,S-TiO_2 and (c) N,S-TiO_2@Ag. Plots of $(\alpha h\nu)^2$ versus $h\nu$ obtained for **(B)** TiO_2, **(C)** N,S-TiO_2 and **(D)** N,S-TiO_2@Ag.

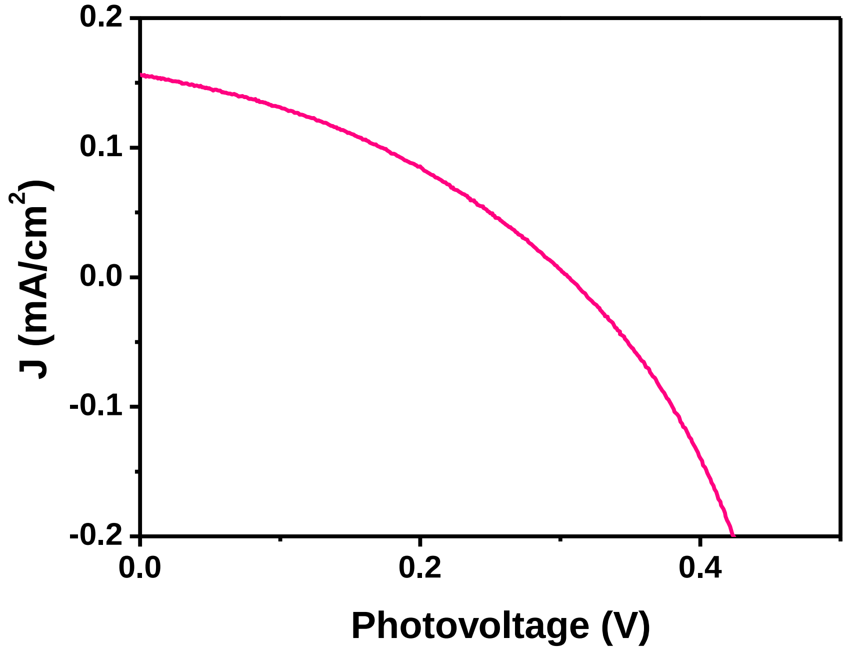

Fig S3 Photocurrent density–photovoltage (*J*–*V*) curves obtained for N,S-TiO_2@Ag nanocomposite thin films in the absence of N719 dye under 100 $mWcm^{-2}$ of simulated AM 1.5G solar light irradiation.

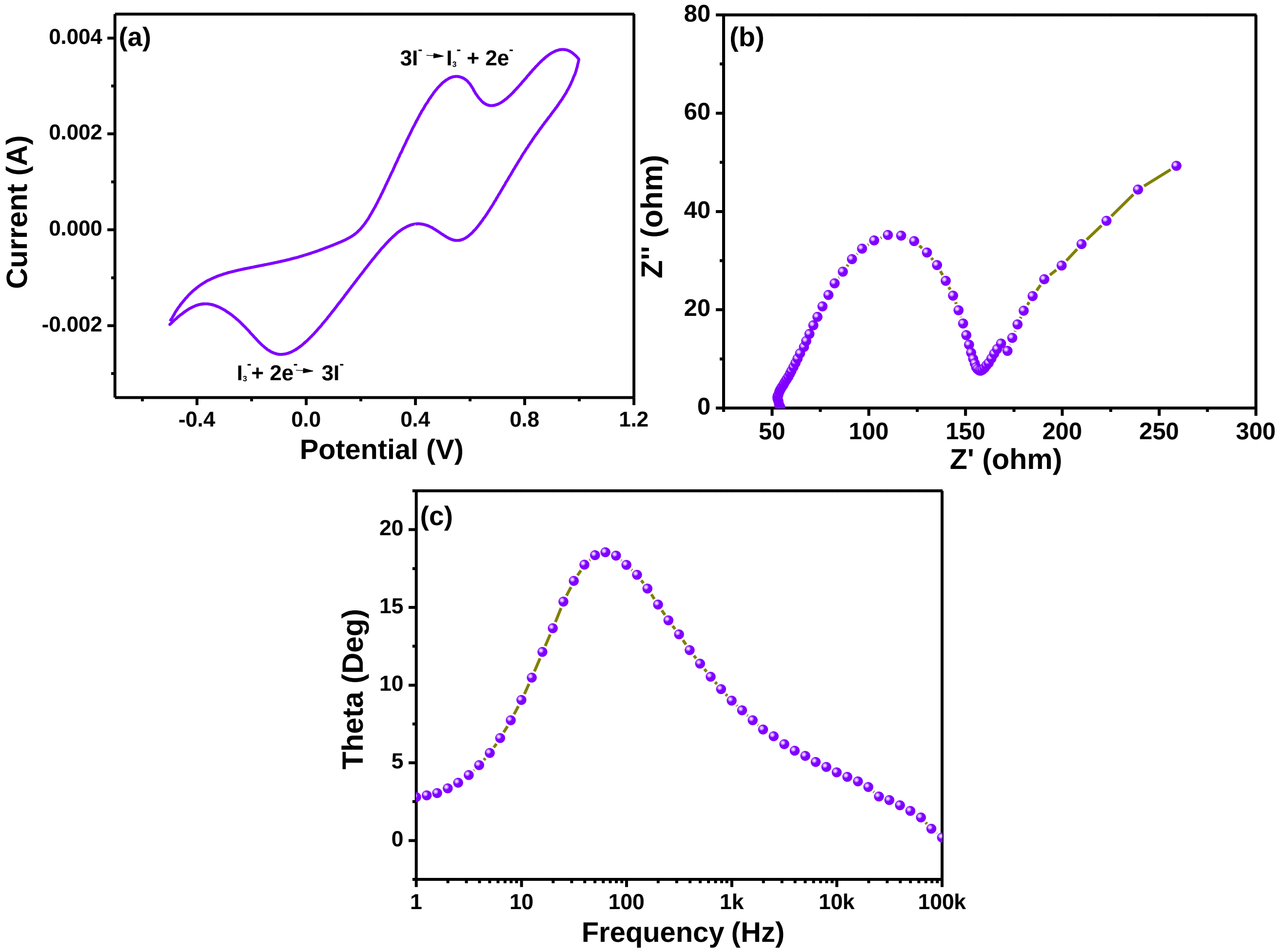

Fig. S4 (a) Cyclic voltammogram (CV), (b) Nyquist and (c) Bode phase plots obtained for Platinum counter electrode in 10 mM LiI, 1 mM I_2 and 0.1 M $LiClO_4$ acetonitrile solution.
